# Early Operations for Hare-Lip

**Published:** 1858-01

**Authors:** 


*Early Operations for Hare-lip.*—Dr. Friedberg relates (Med. Chir. Rev. July, 1856) three cases of hare-lip performed at the following ages, viz : fourteen hours, ten hours, and three hours after birth. Chloroform was administered in each instance, and they all terminated favorably. The same writer advocates the early operation for hare-lip, in his recent work, Chirurgesche Klinik.

To these cases we may add the following: Dr. Dawson (Dub. Med. Press 1842) operated on a child having a simple hare-lip, seven hours after birth. The pins were removed in forty-eight hours, and in two days more the union was so perfect that the mother, who had not seen her child, did not believe any deformity had existed. Mr. Bateman operated (Med.

Times and Gaz. 1854) four hours after birth, where there was also fissure of the palate, so great as to admit the mother's thumb. The operation suceeded, and the fissure of the palate contracted so as scarcely to admit the edge of a sheet of writing paper. Malgaigne advocates early operation, and has operated nine hours after birth. P. Dubois and Guersant prefer operating immediately after birth.—*Va. Med. Journal.*

